# Role of nutritional intervention in patients treated with radiotherapy for pelvic malignancy

**DOI:** 10.1038/sj.bjc.6601868

**Published:** 2004-05-25

**Authors:** C McGough, C Baldwin, G Frost, H J N Andreyev

**Affiliations:** 1 Department of Medicine and Therapeutics, Imperial College Faculty of Medicine, 4th Floor, Chelsea & Westminster Hospital, 369, Fulham Road, London SW10 9NH, UK; 2 Department of Nutrition and Dietetics, Hammersmith Hospitals NHS Trust, Du Cane Rd, London W12 0HS

**Keywords:** pelvic radiotherapy, pelvic malignancy, diet, radiation-induced gastrointestinal toxicity, diarrhoea, nutritional status, enteral/parenteral nutrition

## Abstract

Up to 12 000 patients with gynaecological, urological and rectal cancer undergo radical pelvic radiotherapy annually in the UK. More than 70% develop acute inflammatory changes causing gastrointestinal symptoms during treatment because healthy bowel tissue is encompassed in the radiation field. In total, 50% go on to develop chronic bowel symptoms, which affect quality of life due to permanent changes in the small and large intestine. Nutritional intervention may influence acute and chronic bowel symptoms but the validity of the advice given to patients is not clear. To assess the incidence and significance of malnutrition and to examine the efficacy of therapeutic nutritional interventions used to manage gastrointestinal side effects in patients undergoing pelvic radiotherapy and those with chronic bowel side effects after treatment, a critical review of relevant original studies on human subjects was carried out using a specific set of mesh terms in MEDLINE and EMBASE databases and the Cochrane Library in September 2003. Full texts of all relevant articles were collected and reference lists were checked. Sources of grey literature including conference abstracts and web-based information were also reviewed. A total of 36 papers published in peer-reviewed journals between 1966 and 2003 were identified. In all, 14 randomised controlled trials, 12 prospective cohorts, four retrospective, two qualitative, one validation, one pilot study and two case reports were obtained. These included 2646 patients. Eight articles including three conference abstracts and web-based information were found. None of the studies was definitive because of weakness in methodology. No studies could be combined because the interventions and the end points were different. There is no evidence base for the use of nutritional interventions to prevent or manage bowel symptoms attributable to radiotherapy. Low-fat diets, probiotic supplementation and elemental diet merit further investigation.

A total of 11–12 000 patients with gynaecological, urological and rectal cancer undergo radical pelvic radiotherapy annually in the UK. This reflects about 20% of patients diagnosed with pelvic malignancy (Moller *et al*, 2003). More than 70% develop acute inflammatory small intestinal changes (Resbeut *et al*, 1997), leading to gastrointestinal symptoms during treatment partly because healthy bowel tissue is encompassed in the radiation field.

Acute symptoms include diarrhoea, abdominal pain, tenesmus or nausea that usually start during the second or third week of a course of radical radiotherapy and resolve within a fortnight of completion of radiotherapy (Ajlouni, 1999). The incidence of chronic bowel damage is difficult to assess, as patients may be lost to follow-up, may not report any changes to their clinician or may not be identified by scoring systems historically used in clinical trials. In 5–10% of patients, serious gastrointestinal problems may occur (Ooi *et al*, 1999; Denton *et al*, 2000; Nostrant, 2002). These include bowel obstruction, fistulation, intractable bleeding or secondary cancers. A further 6–78% of patients develop less severe symptoms, which nevertheless detrimentally affect quality of life (Kollmorgen *et al*, 1994; Potosky *et al*, 2000; Gami *et al*, 2003). These may include urgency, frequency, faecal incontinence, diarrhoea, steatorrhoea, tenesmus, pain, constipation and weight loss (Andreyev *et al*, 2003). The severity of acute bowel toxicity may predetermine the degree of chronic bowel changes (Donaldson *et al*, 1975). Therefore, early intervention to prevent or reduce acute toxicity may be worthwhile in the long term.

A number of radiotherapy techniques are used to treat cancers within the pelvis. These may influence the dose that is delivered to the tumour and surrounding structures. Radiotherapy damages tissue because energy dissipated from ionising radiation generates a series of biochemical events inside the cell. Free radicals are formed and disrupt DNA, preventing replication, transcription and protein synthesis. When given in combination with chemotherapy, the risk to normal tissues may be enhanced. The small intestine is particularly susceptible to damage because its cells are usually rapidly proliferating, and bile acid and pancreatic enzymes may potentiate damage to the mucosal glycocalyx (Sullivan, 1962; Mulholland *et al*, 1984).

Consideration of nutrition before, during and after radiotherapy to the pelvis may be important for several reasons. Nutritional risk describes patients who are likely to develop malnutrition as a result of their illness, but the prognostic significance of nutritional risk is not clear. Malnutrition *per se* is an independent adverse prognostic factor in many cancers (Bozzetti, 2001). It may occur due to physiological, metabolic, psychological or iatrogenic processes, which exist as a result of malignancy and may affect morbidity, mortality and response to treatment (Argiles and Lopez-Soriano, 1999).

Specific therapeutic nutritional intervention before and during radiotherapy may induce a radio-protective effect for healthy tissues, for example, elemental diet by various mechanisms including attenuation of biliary and pancreatic secretions (McArdle *et al*, 1974, 1985; Pageau and Bounous, 1977; Mester *et al*, 1990) or nutritional intervention may be used for its radio-enhancing effect on malignant tissues, for example, polyunsaturated fatty acids (Conklin, 2002).

Manipulation of habitual diet after radiotherapy may help to reduce or eliminate chronic, undesirable changes in bowel habit once they have occurred. A number of dietetic interventions such as lactose restriction, fat restriction, reduced intake of motility stimulants such as caffeine and a decrease in fibre-containing foods (Classen *et al*, 1998) have been suggested.

This review has two aims. First, to assess the incidence and significance of malnutrition in patients undergoing pelvic radiotherapy and those with chronic bowel side effects resulting from pelvic radiotherapy and second, to examine the efficacy of therapeutic nutritional interventions used to manage gastrointestinal side effects of pelvic radiotherapy.

## METHODS

A search of original literature was carried out using MEDLINE and EMBASE databases from 1966 to May 2003 and the Cochrane Library. Animal data were excluded. Search terms included pelvic radiotherapy, gynaecological cancer, elemental diet, probiotics, lactose, reduced fat, enteral nutrition, parenteral nutrition, radiation-induced bowel damage, radiation enteritis, bowel symptoms and diarrhoea. These terms were used to generate reference listings, which were then examined against inclusion and exclusion criteria, and full texts of relevant papers were retrieved. Reference lists in individual papers were checked to identify other relevant publications. Grey literature including abstracts of radiotherapy and nutrition conferences and UK doctoral theses were searched in order to obtain unpublished work in the area. Finally, searches using recognised search engines such as ‘Google’, ‘Microsoft Network’ and ‘Ask Jeeves’ were carried out on the Internet to identify information disseminated to the general public and health professionals via new media, especially regarding nonconventional or complementary nutrition support.

Trials were included if they had recruited patients with gynaecological, rectal or urological malignancy and measured acute or chronic gastrointestinal toxicity to pelvic radiotherapy, while intervening with nutrition to alleviate side effects and/or assessed nutritional status of patients before the start of or during a course of pelvic radiotherapy.

The primary outcome sought was bowel toxicity as assessed by the Radiation Therapy Oncology Group scoring tool (Cox *et al*, 1995) (Table 1

**Table 1:** RTOG (Radiation Therapy Oncology Group) toxicity criteria

**Toxicity**	**Grade 0**	**Grade 1**	**Grade 2**	**Grade 3**	**Grade 4**
Lower GI including pelvis	No change	Increased frequency or change in quality of bowel habits not requiring medication/rectal discomfort not requiring analgesics	Diarrhoea requiring parasympatholytic drugs (e.g. Lomotil)/mucous discharge not necessitating sanitary pads/rectal or abdominal pain requiring analgesics	Diarrhoea requiring parenteral support/severe mucous or blood discharge necessitating sanitary pads, abdominal distension (flat plate radiograph demonstrated distended bowel loops)	Acute or subacute obstruction, fistula or perforation; GI bleeding requiring transfusion; abdominal pain or tenesmus requiring tube decompression or bowel diversion

) or other surrogate indicators such as stool frequency and consistency, record of use of antidiarrhoeal medications or patient-reported gastrointestinal symptoms. Secondary outcome measures included nutritional status assessed by change in weight, other anthropometric indicators and changes in dietary intake.

Randomised controlled trials were assessed for methodological quality according to the method of randomisation and group allocation. Studies were graded ‘A’ adequate methodology, ‘B’ inadequate methodology and ‘C’ not stated (The Cochrane Library, 2003). Nonrandomised studies were assessed on methodology and sampling strategy but could not be assessed for quality using any validated grading systems.

## RESULTS

A total of 2646 patients in 36 papers and eight sources of grey literature including three conference abstracts and data in non-peer-reviewed journals or the internet, published between 1966 and 2003, were identified. No systematic reviews, 14 randomised controlled trials, 12 prospective cohorts, four retrospective, one validation study, two qualitative, one pilot study and two case reports were retrieved. No papers have been excluded. The papers are summarised in Tables 2

**Table 2:** Prevalence and changes in nutritional status in patients having pelvic radiotherapy

		**Weight change**				**Nutritional status**		**Dietary intake**		**Bowel toxicity**	
		**Before RT**		**After RT**		**During RT**		**During RT**		**After RT**	
**Author**	**Study type**	**Intervention**	**Control**	**Intervention**	**Control**	**Intervention**	**Control**	**Intervention**	**Control**	**Intervention**	**Control**
Bye (1992)	Randomised controlled trial	15% with >5% loss	12% with >5% loss	2.6 kg loss	1.7 kg loss	9% depleted	6% depleted	18 % had a decreased appetite	20% had a decreased appetite	23% diarrhoea	48% diarrhoea
Ferguson (1999)	Validation study	—		—		89% well nourished 11% moderate malnutrition		—		—	
Hulshof (1987)	Prospective cohort	4.1 kg decrease from habitual weight (*P*<0.05)		No change		—		Decreases (*P*<0.05) in fat, fibre and iron		29% using constipating diet	
Pia de la Maza (2001)	Prospective cohort	—		0.9±1.4 kg decrease (*P*<0.05)		Decrease of 1.0±1.4% body fat (*P*<0.05)		—		87% diarrhoea 80% pain	
Stryker (1980)	Retrospective cohort	—		2.91±2.28% weight loss (*P*<0.05) in 10MV APPA patients		83% lost weight 13% gained weight		—		72% >4 stools/day	

, 3

**Table 3:** Dietary modifications during pelvic radiotherapy

**Study**	**Study type**	***n***	**Intervention**	**Bowel toxicity**	
				**Intervention**	**Control**
Brown (1980)	RCT	68	Elemental diet	—	—
Bye (1992)	RCT	143	Low fat; low lactose	1.1 loose stools/week (*P*<0.01)	1.7 loose stools/week
Capirci (2000)	RCT	680	Elemental diet	16% Grade 1	25% Grade 1
				12% Grade 2	27% Grade 2
Chowdury (2002)	RCT	20	Micronutrient supplement	—	
Craighead (1998)	Phase II feasibility pilot	17	Elemental diet	5.9 (3.4–8.3) days of diarrhoea (*P*<0.05)	12.2 (10.2–14.2) days of diarrhoea
Delia (2002)	RCT	190	VSL #3 probiotic	0% Grade 4	21.4% Grade 4
				65.3% Grade 2	23.8% Grade 2
Foster (1980)	RCT	32	Elemental diet	—	
Karlson (1989)	RCT (conference abstract)	21	Low-fat diet	1.6±0.9 bowel movements/day	2.0±1.0 bowel movements/day
Kinsella (1981)	RCT	32	PN	—	
Liu (1997)	Retrospective study	156	Low residue	Majority Grade 1	
Macia (1991)	RCT	93	Protein/calorie supplementation	—	—
Martin (2002)	Double-blind RCT	56	Enzyme capsule	57% moderate bowel symptoms	36% moderate bowel symptoms (*P*=0.01)
Mcardle (1986)	Prospective cohort	56	Elemental diet	—	
Mccarthy (1999)	Prospective cohort	40	Protein/calorie supplementation	—	
Moriarty (1981)	RCT	51	Protein/calorie supplementation	—	
Salminen (1988)	RCT	24	*L. acidophilus* probiotic	18–27% incidence of diarrhoea (*P*<0.01)	80–90% incidence of diarrhoea
Stryker (1986)	RCT	64	Low lactose	—	—
Valerio (1978)	RCT	20	PN	—	—

, 4

**Table 4:** Internet-based information

**Source**	**Recommendation**	**Evidence**	**Conclusion**
*National Cancer Institute* www.cancer.gov	Diet *low in lactose, fat and residue*		
	*Avoid*	It explains that the evidence is not clear but that such a diet can be effective in managing symptoms	No conclusive evidence
	Milk and milk products		
	Whole bran/cereal, nuts and seeds		
	Fried/fatty foods		
	Fresh fruit, raw veg		
	Strong spices/herbs		
	Choc, tea, coffee, caffeinated soft drinks, alcohol		
			
	Ingest foods at room temp		
	Drink 3 l fluid, let carbonated drinks lose their fizz		
	Add nutmeg to decrease gut motility		
	Start low-residue diet on day 1 RT		
			
*Radiation Oncology Online Journal, USA* www.rooj.com	*Low residue diet*		
	Increased fluid intake	No references	It is clear why some but not all of the recommendations are made
	Small, frequent meals		
	Reduce alcohol		
	*Avoid*		
	High roughage foods and raw veg		
	Tobacco (stimulates gut)		
	Food of extreme temps		
	Carbonated drinks → cause gas		
	*Include* high potassium foods		
	Add nutmeg		
			
*HealthCall-UK* www.internethealthlibrary.com	Reduced fat diet	Website refers to papers discussed above	No evidence to make these recommendations
	Live yogurt and fermented milk products		
			
*Bio Concepts-Cancer Update* www.orthoplex.com.au	Vitamin supplementation before commencing treatment to prevent toxicity, including C,E, glutamine, *β*-carotene, adenosine, cysteine and quercetin	No references included on this web page	Some of the suggestions have been investigated in clinical studies
	Anti-inflammatory agents during treatment, that is, DHA/EPA, quercetin, adenosine, bromelain, Vitamins E and C	No recommended doses or methods of administration given	However, no conclusive evidence is available
	Diet supplementation with glutamine, essential fatty acids and probiotics to prevent radiotherapy-induced diarrhoea		
	Supplementing with Coenzyme Q10, acetyl-L-carnitine, lipoic acid and *α*-ketoglutarate to improve mitochondrial function and thus energy		

and 5

**Table 5:** Dietary modifications after pelvic radiotherapy

**Study**	**Study type**	***n***	**Intervention**	**Bowel toxicity**	
				**Intervention**	**Control**
Beer (1985)	Prospective cohort	8	Elemental diet	Steatorrhoea in seven out of eight patients before intervention	
Bosaeus (1979)	Prospective cohort	9	Low-fat diet	—	
Cohen (1985)	Prospective cohort	20	Magnesium	3 days to stop diarrhoea	2–6 weeks to stop diarrhoea
Danielsson (1991)	Prospective cohort	7	Low-fat diet and bile acid sequestrant	Moderate improvement in symptoms in all patients	
Donaldson (1975)	Retrospective	5	Gluten, cow's milk protein free. Low lactose, fat and residue	All cases asymptomatic at 1 year	
El Younis (2003)	Prospective cohort (conference abstract)	9	Vitamin C and E	All symptoms subsided at 6–12 weeks	
Gami (2003)	Qualitative	107	—	—	
Haddad (1974)	Case report	1	Elemental diet	Symptoms resolved on ED	
Henriksson (1995)	Double-blind RCT	40	*L. Lactis probiotic*	—	
Kennedy (2001)	Prospective cohort	20	Vitamin C and E	Diarrhoea, bleeding and urgency resolved after 4 weeks	
Lavery (1980)	Prospective cohort	5	PN	—	
Levitsky (2003)	Case report	1	Vitamin A	Complete regression of pain and symptoms	
Miller (1979)	Prospective cohort	10	PN	—	
Scolapio (2002)	Retrospective	54	PN	—	
Sekhon (2000)	Qualitative	48	—	—	
Silvain (1992)	Prospective cohort	31	PN	—	
Urbancsek (2001)	Double-blind RCT	206	*L. rhamnosus probiotic*	Reduction (*P*<0.05) in symptoms in both groups. *P*>0.05 between groups	

.

### Methodological quality of trials

Approximately half of the papers reviewed were of randomised controlled study design. However, methodology was often weak, with reporting of method of randomisation, concealment of allocation and blinding lacking from many papers (Brown *et al*, 1980; Foster *et al*, 1980; Kinsella *et al*, 1981; Moriarty *et al*, 1981; Stryker and Bartholomew, 1986; Salminen *et al*, 1988; Karlson *et al*, 1989; Bye *et al*, 1992; Chowdhury *et al*, 2002; Delia *et al*, 2002). The choice of randomisation in papers that reported their methodology was adequate in two studies (Urbancsek *et al*, 2001; Martin *et al*, 2002). Intention-to-treat analyses were described in two papers (Urbancsek *et al*, 2001; Martin *et al*, 2002). It was unclear as to whether such methods were used in other studies. In view of these problems, it is difficult to draw clear conclusions regarding efficacy or effect of the interventions used.

### Malnutrition and pelvic radiotherapy

Five papers (one randomised controlled trial (Bye *et al*, 1992), two prospective cohort (Hulshof *et al*, 1987; Pia de la Maza *et al*, 2001), one retrospective (Stryker and Velkley, 1980) and one validation study (Ferguson *et al*, 1999)) were identified, which assessed the incidence of nutritional risk in patients undergoing pelvic radiotherapy (Table 2). No papers were found that examined whether nutritional status at the start of radiotherapy had any impact on toxicity or outcomes after radiotherapy or the importance of its presence or absence specifically in patients exposed to irradiation to the pelvis. None of the papers assessed the impact of acute diarrhoea on nutritional status, although four of the five identified diarrhoea as an important side effect of treatment, with incidence ranging from 6 to 87%.

Two of the studies (including 121 patients) assessed nutritional risk before starting radiotherapy (Ferguson *et al*, 1999; Pia de la Maza *et al*, 2001). The reported incidence ranged from 11 to 33%. Data were based on patient reports of decreased appetite and weight. In total, 5% weight loss before starting treatment was reported to have occurred in 32% of patients (mean percentage, intervention and control groups) by the randomised controlled trial (Bye *et al*, 1992). The remaining papers (Stryker and Velkley, 1980; Hulshof *et al*, 1987; Bye *et al*, 1992) (380 patients) assessed change in nutritional status during pelvic irradiation. The incidence of weight loss during treatment varied from 0 to 83% in these studies.

### Nutritional interventions

#### Dietary modifications during pelvic radiotherapy

In total, 18 studies were identified that examined dietary interventions in adults and children receiving pelvic radiotherapy (Table 3). The search identified studies comparing a range of nutritional interventions:

- Low-fat diets with or without additional medium-chain triglyceride supplementation compared with unrestricted fat intake or low-fat diets (Karlson *et al*, 1989; Bye *et al*, 1992).
- Lactose restriction or modification either uncontrolled (Stryker and Bartholomew, 1986; Bye *et al*, 1992) or compared to normal diet.
- Low residue diet (Liu *et al*, 1997).
- Probiotic supplementation in sachet or fermented yogurt presentation and modified food intake compared with modified food intake alone (Salminen *et al*, 1988; Delia *et al*, 2002).
- Elemental diet as a supplement to modified food intake or as a sole source of nutritional intake compared to modified diet or total parenteral nutrition (Brown *et al*, 1980; Foster *et al*, 1980; McArdle *et al*, 1986; Craighead and Young, 1998; Capirci and Polico, 2000).
- Enteral and parenteral protein-calorie nutrition support (Valerio *et al*, 1978; Kinsella *et al*, 1981; Moriarty *et al*, 1981; Macia *et al*, 1991; McCarthy and Weihofen, 1999; Chowdhury *et al*, 2002).
- Enzyme preparation supplement (Martin *et al*, 2002).

Low-fat dietary regimens, using 20–40 g fat per day (Karlson *et al*, 1989; Bye *et al*, 1992), induced a significant reduction in diarrhoea, the use of diarrhoea rescue medication and frequency of bowel motions in the 164 patients studied. However, the two studies introduced additional dietary manipulations and did not control for these, which included the use of a medium-chain triglyceride supplement providing 1000 kcal (Karlson *et al*, 1989) and lactose restriction (Bye *et al*, 1992), rendering it unclear as to which intervention had the beneficial effect. Another study focused on lactose, using a randomised controlled design that only modified lactose intake (Stryker and Bartholomew, 1986; Bye *et al*, 1992). No change in bowel symptoms assessed by the RTOG tool were measured in this study (Stryker and Bartholomew, 1986).

A retrospective study assessed the efficacy of introducing a reduced residue regimen in men with prostate cancer undergoing pelvic radiotherapy. It did not identify statistically significant changes in radiotherapy-induced toxicity, particularly gastrointestinal symptoms (Liu *et al*, 1997). In total, 17% of the patients did not comply with the recommended diet.

Two randomised studies, including 214 patients (Salminen *et al*, 1988; Delia *et al*, 2002), used probiotics during pelvic radiotherapy and demonstrated a decrease in the mean number of bowel movements (*P*<0.05) and a decrease in the incidence of diarrhoea (*P*<0.01), using VSL #3 sachets three times daily and 2 × 10^9^ daily dose of a *L. acidophilus* in a fermented yogurt product, respectively. In addition, one of the studies (Salminen *et al*, 1988) also restricted fibre, fat and obvious sources of lactose in all patients.

Five studies (including 847 patients), of which four were randomised controlled trials (Brown *et al*, 1980; Foster *et al*, 1980; McArdle *et al*, 1986; Capirci and Polico, 2000) and one was a phase II pilot study (Craighead and Young, 1998), investigated the use of elemental diet during pelvic radiotherapy. The type of elemental diet implemented varied between studies in terms of the specific product and the relative caloric contribution it provided.

Three studies including a total of 749 patients found a statistically significant decrease in the incidence and severity of acute diarrhoeal symptoms (McArdle *et al*, 1986; Craighead and Young, 1998; Capirci and Polico, 2000). However, the largest study, a multicentre 674 patient trial, has been published only as a conference abstract and a non-peer-reviewed summary booklet. Two of the studies (Craighead and Young, 1998; Capirci and Polico, 2000) used elemental diet as a supplement to normal diet, providing approximately 900 kcal per day. The feasibility study carried out in 17 patients indicated that compliance (deemed as achieving the target volume of elemental diet for more than 80% of the time) to the regimen was achieved in 76.5% of the participating patients. One study (McArdle *et al*, 1986) used elemental diet as the sole source of nutrition in tube-fed patients. The authors revised their methodology and halted randomisation to the parenteral nutrition arm partway through the study. Instead, retrospective controls were used for comparison. They reported a significant perceived benefit in the elementally fed intervention arm. No objective measures were described. Finally, one study (Brown *et al*, 1980) used an elemental-supplemented regimen, but failed to show any significant differences in bowel symptoms. Controls were asked to follow a low roughage diet, while the treatment group followed the same low roughage diet supplemented with three sachets of ‘Vivonex HN elemental feed’ providing 900 kcal. More than 50% of patients could not manage to consume the Vivonex HN for the whole duration of their radiotherapy.

A study comparing a low-fibre diet (specific content unknown) in controls, with the same diet alongside elemental supplementation (Foster *et al*, 1980), did not assess the effect of this intervention on gastrointestinal symptoms. Instead, haematological parameters and weight were compared. There were no significant differences in weight loss between groups.

Four randomised controlled trials (including 204 patients) investigated enteral nutrition support during pelvic radiotherapy (Moriarty *et al*, 1981; Macia *et al*, 1991; McCarthy and Weihofen, 1999; Chowdhury *et al*, 2002). There is little detailed information about the clinical effect that this approach had in terms of treatment toxicity. A range of isocaloric, high protein/calorie enteral supplements were used in two studies (Moriarty *et al*, 1981; McCarthy and Weihofen, 1999) and concluded that the energy and protein intakes of supplemented groups were improved compared to controls. No outcome measures such as toxicity from radiotherapy, tumour control or survival were reported and no significant changes in biochemical or haematological parameters were found.

A study using specific dietary advice to remove gluten and lactose and providing high calorie advice for patients with low appetites (Macia *et al*, 1991) showed a significant decrease in Body Mass Index and Mid-Arm Muscle Circumference in control group patients, but both groups had similar gastrointestinal toxicity.

Two studies, both randomised (Valerio *et al*, 1978; Kinsella *et al*, 1981), evaluated the use of parenteral nutrition *vs* oral nutrition in patients undergoing pelvic radiotherapy. Both indicated that the side effects of treatment and nutritional status were improved in the parenteral fed arms. A reduction in bulk of tumour by 50% was reported in 45% of the parenteral nutrition group (Valerio *et al*, 1978). However, in both studies, group allocation methods meant that there was a strong bias towards severely malnourished patients entering the parenteral nutrition arm.

### Weight changes

**Figure 1:**
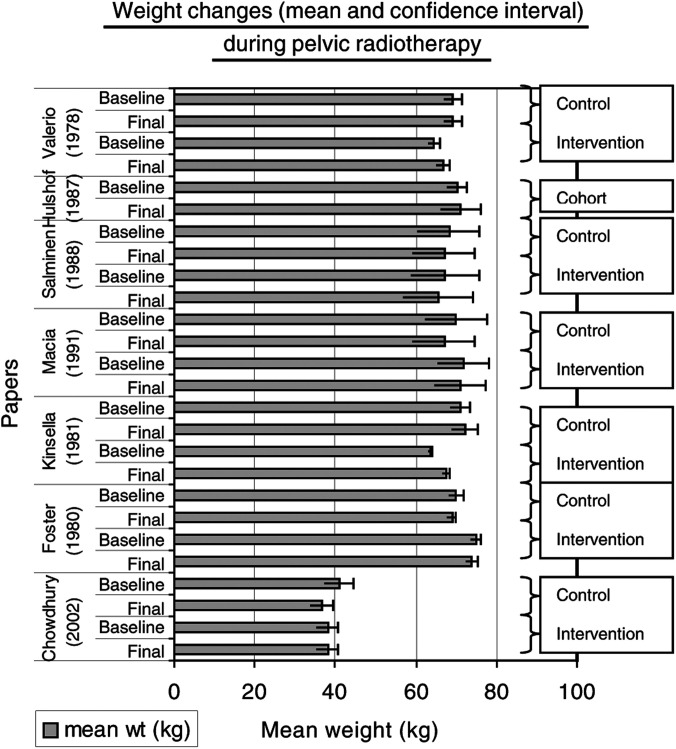
Weight changes and pelvic radiotherapy. (A chart depicting changes in actual weight from start to end of pelvic radiotherapy. A comparison between control and intervention groups is shown.)

**Figure 2:**
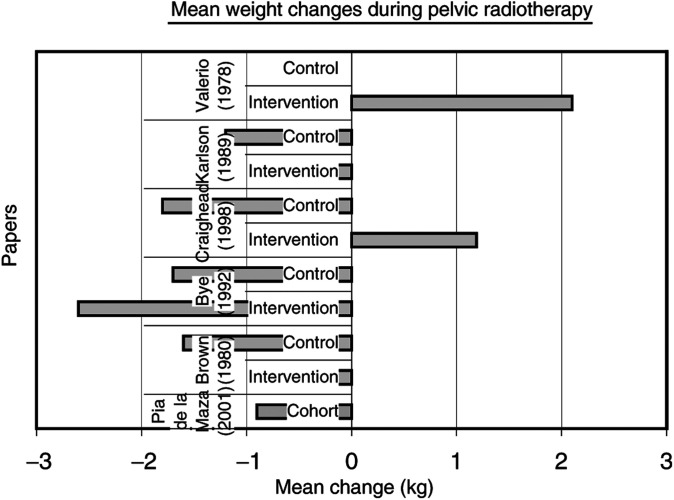
Mean change in weight during pelvic radiotherapy.

Six papers (Valerio *et al*, 1978; Foster *et al*, 1980; Kinsella *et al*, 1981; Salminen *et al*, 1988; Macia *et al*, 1991; Chowdhury *et al*, 2002) recorded changes in actual body weight during pelvic radiotherapy and these data have been combined and displayed as mean weight in kilograms at baseline and completion of radiotherapy for intervention and control arms. Confidence intervals have been calculated (Figure 1

). The mean weight change in kilograms between the start and end of pelvic radiotherapy was recorded in seven papers (Valerio *et al*, 1978; Brown *et al*, 1980; Karlson *et al*, 1989; Bye *et al*, 1992; Craighead and Young, 1998; Capirci and Polico, 2000; Pia de la Maza *et al*, 2001) (Figure 2

).

### Anecdotal dietary recommendations

Advice regarding diet during pelvic radiotherapy was commonly related to restriction of fibre, fat and lactose (National Cancer Institute, Radiation Oncology Online Journal, and Healthcall-UK). Less common suggestions included supplementation with a wide range of micronutrients, coenzymes or amino acids (Bio Concepts-Cancer Update website). Other recommendations included avoiding spicy foods, carbonated drinks and food or drink consumed at extremes of temperature. None of these recommendations were referenced (Table 4).

### Complementary nutrition

One double-blind randomised controlled trial was identified (Martin *et al*, 2002). It assessed the efficacy of introducing an enzyme supplement (WOBE-MUGOS – 100 mg papain, 40 mg chymotrypsin and 40 mg trypsin) available in Germany. Three capsules were taken four times each day. On a diarrhoea scale of 0–3 (0 to >6 bowel movements per day), 43% of the intervention and 64% of the control group experienced only mild symptoms during pelvic radiotherapy. In all, 57% of the intervention and 36% of the control group were rated as having moderate or severe bowel symptoms (*P*=0.11). The Bristol Cancer Help Centre website provided some information regarding diet during radiotherapy. In addition to their controversial restrictive dietary advice aimed at all patients with cancer, there was specific advice to reduce fibre while having radiotherapy to the pelvis.

#### Dietary modifications after pelvic radiotherapy

In total, 17 studies examined the use of dietary modification after pelvic radiotherapy to help to reduce or resolve existing postirradiation gastrointestinal symptoms (Table 5). The nutritional interventions included:

- Probiotic supplementation in fermented milk or sachet presentation compared to placebo (Henriksson *et al*, 1995; Urbancsek *et al*, 2001).
- Elemental diet (uncontrolled) (Beer *et al*, 1985).
- Low-fat diet (uncontrolled) or low fat with bile acid sequestrant (Bosaeus *et al*, 1979; Danielsson *et al*, 1991).
- Gluten and cow's milk protein free with additional lactose and reduced fat/residue (Donaldson *et al*, 1975).
- Parenteral nutrition support (Haddad *et al*, 1974; Miller *et al*, 1979; Lavery *et al*, 1980; Silvain *et al*, 1992; Scolapio *et al*, 2002).
- Reduction of high-fibre foods (Sekhon, 2000; Gami *et al*, 2003).
- Vitamin A, vitamins C and E and magnesium micronutrient therapy (Cohen and Kitzes, 1985; Kennedy *et al*, 2001; El Younis and Abulafia, 2003; Levitsky *et al*, 2003).

Probiotics were used in two double-blinded randomised studies (Henriksson *et al*, 1995; Urbancsek *et al*, 2001), which included 246 patients. These were supplemented into diet as 300 ml twice daily of a fermented yogurt product containing active L1A *Lactobacillus lactis* and one *Lactobacillus rhamnosus* sachet three times daily, respectively. Neither study identified significant improvements in chronic bowel symptoms in patients randomised to the intervention. In one trial (Henriksson *et al*, 1995), gastrointestinal symptoms improved in both groups. However, the control group was taking a placebo probiotic supplement containing strains thought not to be relevant for gastrointestinal symptoms.

Elemental diet as a complete source of nutrition was investigated in a crossover study to manage chronic diarrhoea after pelvic radiotherapy in a group of five malnourished patients from a cohort with chronic pelvic radiation complications (Beer *et al*, 1985). A decrease in faecal weight and the absence of abnormal hydrogen breath tests were reported. Diarrhoeal symptom scores were not measured. A case report (Haddad *et al*, 1974) using exclusive long-term oral elemental diet (27 kcal kg^−1^ day^−1^ with 8% medium chain triglyceride) to treat a patient with abdominal distension, malabsorption and pain showed complete resolution of symptoms while the patient consumed this diet.

Low-fat dietary regimens were used in two studies (Bosaeus *et al*, 1979; Danielsson *et al*, 1991), which included 187 patients. A significant reduction in bile salt malabsorption using a 40 g fat^−1^ day^−1^ diet in nine patients was reported (Bosaeus *et al*, 1979). The other study (Danielsson *et al*, 1991) observed only a moderate improvement in symptoms with the use of a bile acid sequestrant in addition to a low-fat diet.

A gluten-free, cow's milk protein-free, low-residue and low-fat diet was implemented in children with severe radiation enteritis following pelvic irradiation (Donaldson *et al*, 1975). The five case reports suggested that malabsorption and overall nutritional status could be improved with this dietary intervention.

Four cohort studies in patients with chronic, intractable bowel damage after radiotherapy (Miller *et al*, 1979; Lavery *et al*, 1980; Silvain *et al*, 1992; Scolapio *et al*, 2002) assessed parenteral nutrition support in a total of 100 patients. Cyclical nocturnal parenteral nutrition was unsuccessful in controlling severe radiation enteritis symptoms in 48% of the patients (Silvain *et al*, 1992). Nutritional status improved in a small cohort (Lavery *et al*, 1980). Parenteral nutrition was administered for 6–30 months and once weight had stabilised, a mean increase of 12.9 kg was reported. This is in agreement with a similar cohort study (Miller *et al*, 1979), which reported a 60% survival rate at 1 year with a mean weight gain of 8.7 kg (−2.1 to 15). A retrospective study indicated that cumulative survival in patients supported by home parenteral nutrition was 76% at 1 year. There were no comments regarding whether any of the symptoms attributed to radiation bowel damage changed over that period.

Relevant qualitative research was also identified (Sekhon, 2000; Gami *et al*, 2003). Two studies assessed self-imposed changes to dietary intake made by patients with bowel discomfort after radiotherapy. More than 50% of women with chronic bowel change reported increased stool frequency with consumption of bran, pulses and nuts. In 107 patients (Gami *et al*, 2003), no dietary manipulation gave consistent benefit, except for 14 out of 15 patients who eliminated or reduced intake of uncooked vegetables from their diet and reported that bowel symptoms had improved.

The use of micronutrient supplementation in patients with proctitis and other large bowel damage resulting from pelvic radiotherapy has been reported in three studies (Cohen and Kitzes, 1985; Kennedy *et al*, 2001; Levitsky *et al*, 2003) and one conference abstract (El Younis and Abulafia, 2003) with a combined total of 50 patients. An oral dose of 8000 IU vitamin A twice daily administered over 7 weeks is described in a case report (Levitsky *et al*, 2003). All pain and clinical signs of anal ulceration resolved after this intervention. Therapeutic doses of vitamin C (500 mg three times daily) and vitamin E (400 IU three times daily) in combination have been used in two studies to treat radiation proctitis (Kennedy *et al*, 2001; El Younis and Abulafia, 2003). Statistically significant improvements in patient-reported symptoms of bleeding, diarrhoea and urgency, but not pain, were noted and of those patients followed to 1 year, symptom regression was sustained (Kennedy *et al*, 2001). The other study reported all symptoms subsiding by 6–12 weeks of treatment (El Younis and Abulafia, 2003). Finally, a small cohort study described rapid resolution of diarrhoeal symptoms in patients with hypomagnesaemia and radiation-induced proctosigmoiditis with intravenous infusion of magnesium sulphate over 3 days, compared to delayed response on a low-residue diet and use of antidiarrhoeal medication (Cohen and Kitzes, 1985).

## DISCUSSION

This review suggests that the incidence of malnutrition in patients about to start pelvic radiotherapy is 11–33%. Up to 83% of patients lost weight during treatment. Low-fat diets, probiotic supplementation and elemental diet may be beneficial in preventing acute gastrointestinal symptoms. The evidence for the use of nutritional intervention to manage chronic gastrointestinal symptoms is limited. The use of low-fat diets, therapeutic doses of antioxidant vitamins and probiotic supplementation may be helpful. A reduced intake of raw vegetables and fibrous foods may also be effective.

While these conclusions are based on rather weak evidence, they are supported by findings in other disease states. The use of elemental diets to induce remission in Crohn's disease is well established (O'Morain *et al*, 1984; Saverymuttu *et al*, 1985; Gorard *et al*, 1993). Acute radiation bowel damage is also characterised by an inflammatory response. The fat composition of an enteral feed may be important in achieving remission in Crohn's disease (Griffiths *et al*, 1995; Bamba *et al*, 2003). Enteral feeds containing higher proportions of medium-chain triglycerides and n-3 long-chain fatty acids have been reported in studies to infer favourable outcomes when compared with n-6 long-chain fatty acids. This is probably due to their role in the production of eicosapentaenoic acid (EPA) and docosahexaenoic acid (DHA), abundant in fish oils, which have anti-inflammatory effects as opposed to n-6 fatty acids, precursors of arachidonic acid, the substrate for inflammatory eicosanoids (Gorard, 2003). Either this mechanism or its role in reducing the metabolic workload of the gut, or its effects on bile acid or pancreatic enzyme secretion, may explain why elemental diet could be helpful during radiotherapy. Further detailed study is required.

There is also a rationale for the beneficial effect of probiotics in radiation-induced damage. Pathogenic bacterial colonisation can increase the severity of radiation-induced diarrhoea (Urbancsek *et al*, 2001). Re-colonisation with an optimal species could attenuate such an effect (Urbancsek *et al*, 2001). Probiotic bacteria can also signal with the gastrointestinal epithelium via mucosal regulatory T-cells to modulate intestinal inflammation (Caradonna *et al*, 2000). Lactose intolerance secondary to an inflamed mucosa can be resolved using probiotic bacteria, which can potentially ferment luminal lactose to prevent osmotic diarrhoea from occurring.

Finally, ionising radiation is a pro-oxidant process and creates free radicals. Antioxidant vitamins A, C and E may have a synergistic effect in scavenging reactive oxygen species and play a beneficial role in the molecular mechanism of ischaemic injury in the gut (Empey *et al*, 1992). For these reasons, supplementation with therapeutic doses to patients with chronic radiation bowel damage, which is a vascular, non inflammatory process, may improve clinical symptoms (Thomson *et al*, 1998).

There is a scientific basis for studies of nutritional intervention in humans. A large number of animal experiments have identified potential physiological mechanisms occurring in the small and large intestine during and after pelvic radiotherapy. Interventional studies using elemental diet, micronutrient supplementation and probiotics suggest that some of these physiological mechanisms can be blocked, leading to significant reduction in radiation damage (Hugon and Bounous, 1972, 1973; Bounous *et al*, 1973; Pageau *et al*, 1975; Pageau and Bounous, 1977; McArdle *et al*, 1985, 1986; McArdle, 1994; Wiseman *et al*, 1996; Mutlu-Turkoglu *et al*, 2000).

The primary aim of nutritional intervention should be to show benefit in relevant outcomes using adequate tools to measure gastrointestinal toxicity. Most published studies have failed to do this either because of inadequacies in their methodology or because they fail to report important end points.

There are many key questions that remain to be answered. What physiological changes occur in the human gastrointestinal tract when the pelvis is irradiated? How significant are such changes? Can a specific nutritional intervention given during pelvic radiotherapy modulate individual physiological changes? Does this prevent the onset or reduce the severity of clinically occurring gastrointestinal symptoms? When should nutritional intervention be given? How should it be given? Which formulations would enable compliance? Which patients would benefit from intervention?

To begin to answer these questions, well-designed randomised studies are needed. Health professionals working with these patients who may not be trained in nutrition will need to adopt a multidisciplinary approach to research. Patients need to consent to participate in randomised studies in which they may not receive the perceived ‘beneficial’ intervention. This is difficult at a time of high anxiety and uncertainty as a result of their diagnosis. Finally, appropriate end points using established, comprehensive, validated assessment techniques must be incorporated in studies that are large enough to answer the questions asked, to ensure that the results obtained are meaningful in relation to clinical practice.

Gastrointestinal symptoms induced by pelvic radiotherapy can cause morbidity and distress in the acute phase during treatment and can also develop into a chronic, intractable form months or years after the cessation of treatment (Denton *et al*, 2002). Increasingly, patients are being treated successfully and curatively (UKCCCR Anal Cancer Trial Working Party, 1996). However, if life expectancy is increased then it is even more crucial to ensure that an individual patient's quality of life remains high and is not detrimentally affected by the very treatment that has saved their life. To conclude, it is imperative that well-designed randomised controlled studies are carried out to evaluate the nutritional interventions that have been identified by current literature as having potential benefit in patients treated with pelvic radiotherapy.
